# A Hospital Pageant?

**Published:** 1921-05-07

**Authors:** 


### The Hospital

The Workers' Newspaper of Administrative Medicine and Institutional Life, Administration, National Insurance and Health.

No. 1822, Vol. LXX. SATURDAY, MAY 7, 1921. PRICE TWOPENCE

### A HOSPITAL PAGEANT?

It will be remembered that when in our issue of April 16 the question of a general effort to obtain support for hospitals was examined it was promised that this journal would give of its best to aid such a movement. The Hospital now makes a practical suggestion, which it is hoped will commend itself to those of our hospital managers who wish to be up and doing in the necessary task of interesting the multitude.

It is estimated that 90 per cent. of the population of the Metropolis, if not of the country, know little or nothing about the hospitals, what they are doing, or even where they are situated, except the few institutions which have had the fate to find themselves, more by accident than design, in a much-frequented thoroughfare. None can deny that collective publicity and propaganda constantly carried out, therefore, are necessary to bring home the knowledge of hospitals and their work in order to secure the support of the many.

Hospital Sunday is approaching. We propose that on the preceding day there should take place a Hospital Pageant and Carnival, taking the form of a procession through the principal thoroughfares of London. Those hospitals which are on the outskirts of London are familiar with what is known as a "Demonstration." What we suggest is a Super-Demonstration which will attract the attention, and we hope the support, of a very large number of persons, many of whom may not attend places of worship on Hospital Sunday. The proceeds, of course, would go to swell the takings of that popular festival.

Hospital secretaries have usually something of the showman in their make-up, and this attribute is becoming more and more essential to the needs of the institutions they serve. These gentlemen, if they got together to discuss our proposals—Mr. Panter of the Great Northern Central Hospital, or Mr. Coles of King's College Hospital, both advertising experts, might convene a meeting—would quickly develop the idea we venture to bring forward. They would find that an ample number of workers on such an occasion would not be wanting. The students of the teaching hospitals and, should the authorities agree, possibly also the nurses would make sturdy advocates or helpers of the cause.

Business firms, whether supplying hospitals or not, would gladly lend vehicles to bear the tableaux and exhibits of the moving exhibition, which would "feature," to use a cinema term, the *x*-ray room, the operating-theatre, and the ward. A graduated series of pictures would teach the history of hospitals, from the monkish healing of the Middle Ages to the perfected methods of the present day. The press, which has been so helpful throughout the hospital crisis, would, we are sure, lend its weighty aid to prepare London for the coming event. For our part we are open to produce a special supplement to The Hospital, to appear concurrently with the pageant, containing facts and figures having a distinct educative value which also will be found useful for reference by the clergy on the following day.

These are but suggestions to be elaborated at the will of a strong committee of hospital workers, whose experience and knowledge are enough to secure success. All are agreed that the hospital appeal must be pushed far beyond the ground that has sufficed in the past to provide the needs of the voluntary hospitals. We hope that our suggestion will result in an opportunity of approaching the citizens of London in a way which has never been attempted before.

